# Vitellogenin Functions as a Multivalent Pattern Recognition Receptor with an Opsonic Activity

**DOI:** 10.1371/journal.pone.0001940

**Published:** 2008-04-09

**Authors:** Zhaojie Li, Shicui Zhang, Qinghui Liu

**Affiliations:** 1 Department of Marine Biology, Ocean University of China, Qingdao, People's Republic of China; 2 Yellow Sea Fishery Research Institute, Chinese Academy of Fisheries, Qingdao, People's Republic of China; University of Birmingham, United Kingdom

## Abstract

**Background:**

Vitellogenin (Vg), a major reproductive protein, has been associated with infection-resistant response in fish. However, the underlying mechanisms by which Vg is involved in anti-infectious response are not understood.

**Methodology/Results:**

By both protein-microbe interaction analysis and enzyme-linked immunosorbent assay as well as phagocytosis test, we demonstrate for the first time that fish Vg acts as a pattern recognition molecule with multiple specificities that can recognize bacteria as well as fungus rather than self components from fish, and functions as an opsonin that can enhance macrophage phagocytosis.

**Conclusions:**

This study shows that fish Vg plays an integrative function in regulating immunity via its pleiotropic effects on both recognizing pathogen-associated molecular patterns and promoting macrophage phagocytosis. It also supports the notion that factors normally involved in control of female reproduction are associated with immunity in organisms that rely on Vg for oocyte development.

## Introduction

Vitellogenin (Vg), a phospholipoglycoprotein, is a member of the lipid transfer protein superfamily including apolipoprotein B (apoB), microsomal triglyceride transfer protein and Vg [1], and constitutes the precursor of major yolk proteins in all oviparous organisms. It is usually synthesized by the extraovarian tissues of female animals, secreted into bloodstream and transported to ovary, where it is internalized by growing oocytes and proteolytically cleaved to form yolk proteins that are later used as the nutrients by developing embryos and larvae [2]–[7]. However, Vg appears to have evolved pleiotropic functions in the advanced eusocial honeybee. It has been shown that honeybee Vg is associated with quite a few biological processes including social organization, temporal division of labor and foraging specilization, regulation of hormonal dynamics, and change in gustory responsiveness [8]–[11]. Besides, honeybee Vg has been shown to be capable of reducing oxidative stress by scavenging free radicals, thereby prolonging lifespan in the facultatively sterile worker castes and reproductive queen castes [12]. Similar antioxidant activity has also been observed for nematode (*Caenorhabditis elegans*) Vg [13].

Another novel function of Vg is linked with immune defense [14]. For example, Vg has recently been demonstrated to possess both hemagglutinating and antibacterial activities in the protochordate amphioxus (*Branchiostoma belcheri*) as well as the bony fish rosy barb (*Puntius conchonius*) [15], [16]. Moreover, the male rosy barb is able to produce Vg while challenged with *Escherichia coli*, implicating that Vg may be related to infection-resistant response. However, the mode of action by which Vg is involved in anti-infectious response remains unknown.

Non-self recognition and phagocytosis are two pivotal processes in innate immune response. Recognition of non-self in innate immunity is mediated by a set of germline-encoded proteins known as pattern-recognition receptors [17], [18], that recognize the microbial cell wall constituents called pathogen-associated molecular patterns (PAMPs), such as lipopolysaccharide (LPS) of Gram-negative bacteria, lipoteichoic acid (LTA) of Gram-positive bacteria, and β-1,3-glucan of eukaryotic fungi [18], [19], [20]. Phagocytosis, a crucial defense mechanism against microbial infection, is performed by professional phagocytes such as macrophages and dendritic cells in vertebrates [21]. Humoral proteins such as antibody fragments and complement components from vertebrates can bind to the surface of pathogens and function as opsonins to promote phagocytosis [22], [23]. Scavenger receptors and cell surface C-type lectins also enhance phagocytosis [24]. Similarly, in invertebrates like insects, plasma proteins such as lectins and complement-like factor, and membrane proteins including peptidoglycan recognition protein LC also mediate phagocytosis [25], [26], [27]. Relatively, non-self recognition and phagocytosis have not as yet been given sufficient attention in fish.

The aims of this study were to determine if fish Vg is a pattern recognition molecule, and if so, to examine if it has an opsonic activity to promote macrophage phagocytosis. It is demonstrated for the first time that Vg from fish *Hexagrammos otakii* (Jordan & Starks) can not only act as a multivalent pattern recognition receptor binding to LPS, LTA, peptidoglycan (PGN), glucan and laminarin, but also function as an opsonin promoting macrophage phagocytosis.

## Results

### Purification and characterization of Vg

**Figure 1:**
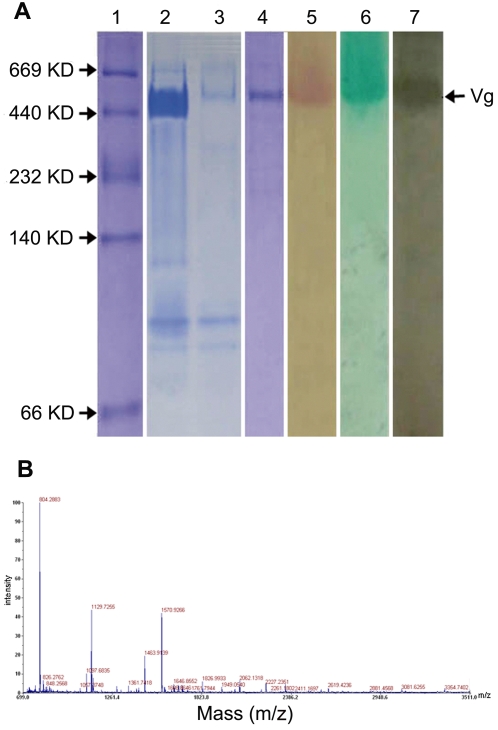
Purification and identification of Vg. (A) Purification of Vg and determination of carbohydrate, lipid and phosphorus components. The proteins were run on a 7.5% native PAGE. lane 1, marker; lane 2, plasma (diluted 20-fold) of fish injected with E_2_; lane 3, plasma (diluted 20-fold) of fish injected with 0.9% NaCl; lane 4, purified Vg; lanes 5, 6 and 7, purified Vg stained with Schiff reagent, methyl green and Sudan black B, respectively. The protein induced by E_2_ stained positive for carbohydrate, lipid, and phosphorus. (B) Peptide mass mapping of the purified protein.

In fishes, Vg is a protein with a molecular weight ranging from 250 kDa to 600 kDa [28], [29]. Compared with untreated fish, plasma from 17-β-estradiol (E_2_)-injected *H. otakii* had an intense band of protein with a molecular mass of ∼450 kDa (Fig. 1*A* lanes 2 and 3), suggesting that it was E_2_-inducible protein. The protein from E_2_-injected fishes was purified by double-step chromatography (Fig. 1*A* lane 4), and the purified protein stained positive for carbohydrate, lipid, and phosphorus (Fig. 1*A* lanes 5, 6 and 7), thus establishing the protein as a phospholipoglycoprotein. Peptide mass mapping analysis performed on an Applied Biosystems 4700 proteomics analyzer further revealed that the protein purified was Vg from fish *H. otakii* (Fig. 1*B*).

### Vg binds to both Gram-negative and Gram-positive bacteria as well as fungus

The F/P ratios of FITC-labeled Vg, apoB and bovine serum albumin (BSA) were 4.9, 6.1 and 5.4, respectively, showing that they had similar F/P ratios. Antibacterial activity examination by colony forming unit assay showed that the growth of *E. coli* pre-incubated with 50 µg/ml of FITC-labeled Vg and unlabeled Vg was significantly inhibited, with the similar inhibitory rates of 27% and 29%, individually. In addition, FITC-labeled Vg appeared as a single band on a native 7.5% native-PAGE, suggesting that FITC-labeled Vg is able to dimerise (data not shown). These indicated that Vg activity is not affected by labeling with FITC.

**Figure 2:**
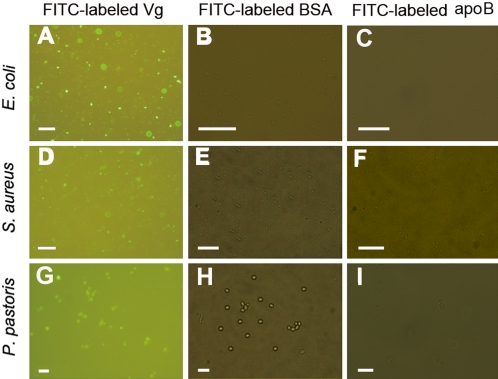
Binding of FITC-labeled Vg to microbial cells. Vg was labeled by FITC, and FITC-labeled Vg was incubated with Gram-negative bacterium *E. coli*, Gram-positive bacterium *S. aureus* and fungus *P. pastoris*, respectively. After washing three times with 25 mM Tris-HCl buffer containing 137 mM NaCl and 3 mM KCl (TBS; pH 7.6), the microbes were harvested by centrifugation, applied to microscope slides, and observed under an Olympus BX51 fluorescence microscope. The microbes treated with FITC-labeled BSA and FITC-labeled apoB were processed under the same conditions. (A, D and G) Binding of FITC-labeled Vg to *E. coli, S. aureus* and *P. pastoris*; (B, E and H) Non-binding of FITC-labeled BSA to *E. coli, S. aureus* and *P. pastoris*; (C, F and I) Non-binding of FITC-labeled apoB to *E. coli, S. aureus* and *P. pastoris*. A, D and G are the images under a fluorescent field, while B, E, H, C, F and H are the merged images under fluorescent and bright field channels. Scale bars: 20 µm.

To test if Vg can bind to microbes, FITC-labeled Vg was incubated with Gram-negative bacterium *E. coli*, Gram-positive bacterium *Staphylococcus aureus* and fungus *Pichia pastoris*. It was found that Vg bound to both *E. coli* and *S. aureus* as well as *P. pastoris* (Fig. 2). In contrast, none of FITC-labeled apoB and FITC-labeled BSA employed as controls bound to the microbial cells tested. These revealed the specificity of Vg binding to the microbes.

### Vg binds to various ligands

**Figure 3:**
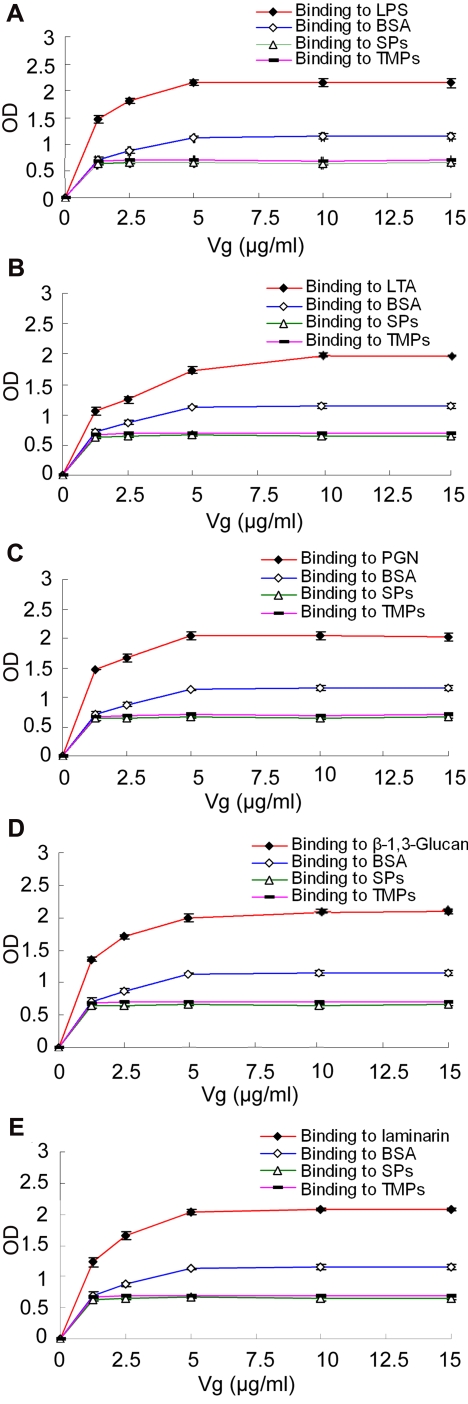
Binding of Vg to various ligands. LPS, LTA, PGN, laminarin and β-1,3-glucan dissolved in re-distilled water were applied to a 96-well microplate, and air-dried overnight at room temperature, followed by ELISA. BSA, serum proteins (SPs) and total muscle proteins (TMPs) of *H. otakii* at the same concentrations were treated similarly as controls. Data were expressed as mean values±SEM (n = 3). The bars represent the standard error of mean values. Although Vg slightly binds to BSA and SPs and TMPs of *H. otakii*, its affinity to the immobilized ligands is significantly higher (*p*<0.05). (A–E) Binding of Vg to LPS, LTA, PGN, β-1,3-glucan and laminarin.

To better understand the mechanisms of binding activity, an enzyme-linked immunosorbent assay (ELISA) was carried out to investigate what molecules on the microbial surfaces are recognized by Vg. Although Vg slightly bound to BSA, it had a significantly stronger affinity to the immobilized ligands including LPS from Gram-negative bacteria, LTA from Gram-positive bacteria, PGN from both Gram-positive and Gram-negative bacteria, β-1,3-glucan from fungi and laminarin from brown algae (*p*<0.05; Fig. 3). Moreover, the affinity of Vg to the serum proteins (SPs) and total muscle proteins (TMPs) extracted from *H. otakii* itself was also markedly lower than that of Vg binding to the immobilized ligands (*p*<0.05). These demonstrated that Vg possesses a significantly higher affinity to the microbial surface molecules rather than BSA and the self components from *H. otakii* itself.

### Vg enhances phagocytosis of microbes *in vitro*

**Figure 4:**
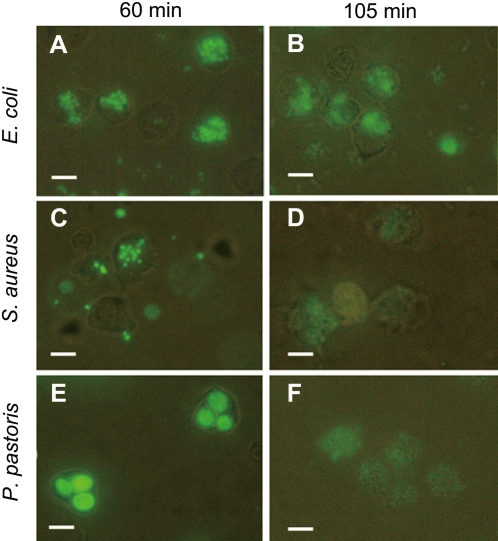
Phagocytosis of *E. coli*, *S. aureus* and *P. pastoris* by macrophages. Vg, FITC-labeled microbes and macrophages freshly isolated were mixed and incubated together at room temperature. The controls were performed in the presence of BSA instead of Vg and in the absence of Vg. Phagocytosis was observed at 60 min after mixing macrophages with microbes. (A, C and E) Phagocytosis of *E. coli*, *S. aureus* and *P. pastoris* by macrophages at 60 min; (B, D and F) Phagocytosis of *E. coli*, *S. aureus* and *P. pastoris* by macrophages at 105 min. Scale bars: 10 µm.

**Figure 5:**
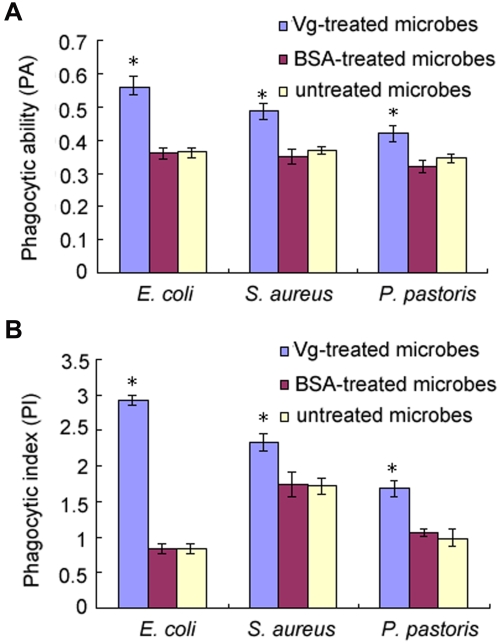
Phagocytic ability (PA) and phagocytic index (PI) of macrophages engulfing *E. coli*, *S. aureus* and *P. pastoris*. Both Vg in PBS and BSA in PBS as well as PBS alone were pre-incubated with the FITC-labeled microbes, *E. coli, S. aureus* and *P. pastoris*, and then incubated with the macrophages freshly isolated. Phagocytosis was observed at 60 min after mixing macrophages with microbes. The microbes pre-incubated with Vg were readily phagocytosed by the macrophages, while those precubated with BSA or PBS alone were not. The PA and PI values of the macrophages phagocytosing Vg-treated *E. coli, S. aureus* and *P. pastoris* were significantly higher than those of the cells phagocytosing BSA-treated and untreated microbes (*p*<0.05). There was no significant difference in the PA and PI values of the cells engulfing BSA-treated and untreated microbes. Data are expressed as mean values±SEM from three experiments. The bars represent the standard error of the mean, and asterisks denote statistically significant differences (*p*<0.05). (A) PA of macrophages engulfing *E. coli, S. aureus* and *P. pastoris.* (B) PI of macrophages engulfing *E. coli, S. aureus* and *P. pastoris.*

The macrophage population isolated from the head kidney (front part of fish kidney) of *H. otakii* had a viability of about 98%. To examine if Vg promotes macrophage phagocytosis, both Vg in PBS and BSA in PBS as well as PBS alone were pre-incubated with the FITC-labeled microbes, *E. coli, S. aureus* and *P. pastoris*, and then incubated with the macrophages freshly isolated. Pilot experiments showed that both phagocytic ability (PA) and phagocytic index (PI) reached maximum values at 60 min after incubation of macrophages with microbes, and the engulfed microbes became digested to pieces at 105 min (Fig. 4). Therefore, phagocytosis was observed at 60 min after mixing macrophages with microbes in subsequent experiments. Compared with the microbes pre-incubated with BSA and PBS alone, those pre-incubated with Vg were readily phagocytosed by the macrophages. The PA and PI values of the macrophages phagocytosing Vg-treated *E. coli, S. aureus* and *P. pastoris* were 56.0±3.5%, 49.0±2.0% and 42.0±2.4%, and 2.920±0.072, 2.333±0.113 and 1.677±0.118, respectively, while the PA and PI values of the cells engulfing BSA-treated and untreated *E. coli, S. aureus* and *P. pastoris* were 36.0±1.7%, 35.0±2.0% and 32.0±1.8%, and 36.3±1.8%, 36.9±1.2% and 34.5±1.3%, and 0.831±0.065, 1.740±0.179 and 1.060±0.049, and 0.836±0.049, 1.719±0.046 and 0.991±0.015, individually (Fig. 5). Apparently, the percent of macrophages phagocytosing Vg-treated microbes was significantly higher than that of cells phagocytosing BSA-treated and untreated microbes (*p*<0.05). Furthermore, the number of Vg-treated microbes engulfed by each macrophage was also significantly higher than that of BSA-treated and untreated microbes engulfed by each macrophage (*p*<0.05). In contrast, there was no significant difference in the PA and PI values of the cells engulfing BSA-treated and untreated microbes. These denoted that Vg is able to promote the macrophage phagocytosis.

### Vg binds to macrophages

**Figure 6:**
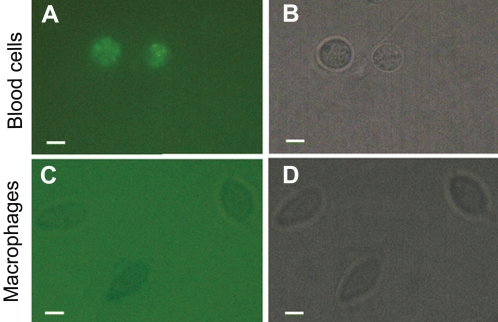
Binding of FITC-labeled Vg to macrophages. Macrophage suspensions and FITC-labeled Vg were incubated together at room temperature for 30 min, and then 60 µl of cell suspensions was sampled to make smears for microscopic examination under an Olympus BX51 fluorescence microscope. For control, red blood cells were used, and treated similarly. A and C are the images under a fluorescent field, and B and D are the images under a bright light field. (A and B) Macrophages. (C and D) Red blood cells. Scale bars: 10 µm.

To test if Vg can preferentially bind to macrophages, FITC-labeled Vg was incubated with the macrophages and red blood cells, respectively. It was found that Vg bound to the surface of macrophages, but not red blood cells (Fig. 6), suggesting that Vg is capable of binding to macrophage surfaces specifically.

## Discussion

Vg, a major reproductive protein, has been proposed to perform pleiotropic functions including immune defense reaction. Our results suggest that fish Vg plays an integrative role in regulating innate immunity via its recognizing microbial cell wall constituents PAMPs and enhancing macrophage phagocytosis.

It is shown for the first time in this study that fish Vg binds to LPS from Gram-negative bacteria, LTA from Gram-positive bacteria, PGN from both Gram-positive and Gram-negative bacteria, β-1,3-glucan from eukaryotic fungi and laminarin from brown algae. In agreement, fish Vg also binds to both Gram-negative bacterium *E. coli* and Gram-positive bacterium *S. aureus* as well as fungus *P. pastoris*. However, Vg fails binding to self molecules including the serum components and total muscle proteins prepared from *H. otakii*. Recently, significant advances have been made in identifying the non-self-recognition molecules, and few of them have been tested for the existence of multiple specificities recognizing PAMPs [30]–[33]. It is clear that fish Vg is a novel pattern recognition receptor with a wide spectrum of specificity capable of identifying non-self components including LPS, LTA, PGN, glucan and laminarin.

It is of particular interest to note that fish Vg can bind to *P. pastoris*, and recognize β-glucan. As far as we are aware, β-glucan binding proteins have been documented only in invertebrates [34]. In contrast, only a few cell surface receptors including dectin-1 and scavenger receptors are known to mediate recognition of β-glucan in vertebrates [35], [36], while no plasma proteins recognizing β-glucan have ever been documented. Therefore, fish Vg appears the first plasma β-glucan binding molecule reported so far in vertebrates.

Plasma proteins that bind to both microbes and surface receptors of macrophages function as opsonins to promote phagocytosis. We show here that in addition to acting as a pattern recognition receptor with multiple specificities, fish Vg is able to bind to the surface of macrophages (Fig. 6) and enhance macrophage phagocytosis (Fig. 5). Enhanced phagocytosis by fish Vg suggests that Vg has an opsonic activity, which is apparently attributable to its ability to bind both microbes and macrophages (Fig. 2 and Fig. 6). Further study is needed to identify the surface molecules on macrophages to which Vg binds.

Overall, it is highly likely that fish Vg is physiologically involved in both the sensing of invading pathogens via interaction with their PAMPs and the recruitment of macrophages to phagocytose and digest the pathogens. The study of *in vivo* function of fish Vg is currently under way.

In summary, the present study highlights previously unreported roles for teleostean Vg in two key areas of fish innate immunity, namely, microbial molecular pattern recognition and macrophage phagocytosis. It also bolsters the notion that factors normally involved in control of female reproduction are associated with immunity in organisms that rely on Vg for oocyte development.

## Materials and Methods

### Purification and characterization of Vg

A total of 12 fishes *H. otakii* at the previtellogenic stage and with an average weight of 500 g were purchased from a local fish dealer, and reared at room temperature in two tanks with 50 L of seawater, which was changed once a day. Six fishes *H. otakii* were each injected intraperitoneally with 2 mg/kg body weight of 17-β-estradiol (E_2_) dissolved in ethanol plus 0.9% NaCl (1∶1, v/v). Two injections were given at 7-days intervals. Another 6 *H. otakii* received two intraperitoneal injections of only ethanol plus 0.9% NaCl. Seven days after the second injection, fish were anaesthetized with 0.05% 3-aminobenzoicacid ethyl ester (Sigma–Aldrich, Germany), and blood samples (totally about 12 ml) were collected via the caudal sinus with heparinized syringes, placed in heparinized tubes containing 0.5 mM phenylmethylsulfonyl flunoride (PMSF, Sigma), and centrifuged at 3,000 g at 4°C for 20 min. Serum was pooled, and Vg was purified as described previously [37].

Native-polyacrylamide gel electrophoresis (PAGE) was carried out on a 7.5% separation gel with a 4% spacer gel using the buffer system of Davis [38]. After electrophoresis, proteins were stained with Coomassie brilliant blue R-250. To test the presence of carbohydrate, lipid and phosphorus components, the gels were stained using periodic acid/Schiff reagent [39], Sudan black B [40] and methyl green [41], respectively. The target protein band was also excised from the gel, and subjected to trypsin digestion and mass spectrophotometry analysis [42].

### Labeling Vg with fluorescein isothiocyanate (FITC)

Purified Vg (1 mg/ml) was adjusted to pH 9.8 by dialyzing against 200 mM carbonate buffered solution (CBS; pH 9.8) at 4°C for 24 h. To label Vg with FITC, Vg treated as above was dialyzed against CBS (pH 9.8) with 0.1 mg/ml FITC (Sigma) at 4°C for 24 h. To separate the FITC-labeled Vg from free FITC, the mixture was dialyzed against 10 mM phosphate buffered solution (PBS; pH 7.4), which was changed twice a day, at 4°C for about 4 days. The purity of the conjugate, FITC-labeled Vg, was confirmed by native-PAGE and Coomassie blue staining, and its F/P ratio (F/P ratio is defined as the ratio of moles of FITC to moles of protein in the conjugate) was calculated by the equation F/P = (2.77×A_495_)/[A_280_-(0.35×A_495_)] from the absorbance readings of the conjugate samples [43]. Bovine serum albumin (BSA) and human apolipoprotein B (apoB), a member of protein belonging to the same superfamily as Vg, were also labeled with FITC using the same method.

To assess if Vg activity is affected by labeling with FITC, the colony forming unit (CFU) assay was conducted to compare the growth inhibition rates of *E. coli* by FITC-labeled Vg and unlabeled Vg [16]. Briefly, an aliquot of 60 µl of FITC-labeled Vg (100 µg/ml) was mixed with 60 µl of *E. coli* suspension with 2×10^4^ cells/ml, and the mixture was pre-incubated, with gentle stirring, at 25°C for 1 h, and plated onto 3 plates (40 µl each plate) with LB media. After incubation at 37°C for 12 h, the resulting bacterial colonies in each plate were counted. The controls were processed similarly except that FITC-labeled Vg was replaced with Vg or PBS alone.

### Assay for binding of FITC-labeled Vg to microbial cells

Binding of Vg to microbes was assayed by the method of Jiang et al. [44] with slight modification. In brief, aliquots of 30 µl of FITC-labeled Vg (0.2 mg/ml) in PBS were mixed with 30 µl of Gram-negative bacterium *E. coli* (5×10^7^ cells), Gram-positive bacterium *S. aureus* (5×10^7^ cells), and eukaryotic fungus *P. pastoris* (5×10^6^ cells), respectively, at 4°C overnight. The microbes were washed three times with 1.0 ml of 25 mM Tris-HCl buffer containing 137 mM NaCl and 3 mM KCl (TBS; pH 7.6), harvested by centrifugation at 3,000 g at room temperature for 15 min, and re-suspended in 1 ml of TBS. Aliquots of 10 µl of the microbial suspensions were applied to microscope slides, and binding of FITC-labeled Vg to the microbial cells was observed under an Olympus BX51 fluorescence microscope. The microbes treated with FITC-labeled BSA and FITC-labeled apoB under the same conditions were used as controls.

### Labeling Vg with DIG

Labeling Vg with DIG was performed according to the instruction of DIG protein labeling kit (Roche). Purified Vg was dialyzed against 10 mM PBS (pH 7.4) at 4°C overnight, and adjusted to a concentration of 1 mg/ml with 10 mM PBS. A total of 1 mg/ml Vg in 10 mM PBS was mixed with 16.35 µl of 20 mg/ml DIG-NHS solution dissolved in dimethylsulfoxide (DMSO; Sigma) and incubated at 25°C for 2 h. The remaining non-reacted DIG-NHS was removed by gel filtration on a Sephadex G-25 column.

### Preparation of serum and total muscle proteins

Fishes *H. otakii* were anaesthetized with 0.05% 3-aminobenzoicacid ethyl ester, and the blood was collected in 10 ml eppendorff tubes from the caudal vein, and the muscle dissected out. The blood samples were allowed to clot at room temperature for 1 h, placed at 4°C overnight, and the sera pooled by centrifugation at 200 g for 10 min.

Total muscle proteins (TMPs) were prepared by the method of Chen and Resh [45]. In brief, the muscle tissue was triturated in liquid nitrogen with mortar, lysed in RIPA buffer (150 mM NaCl, 1 mM EDTA, 0.1% SDS, 0.5% deoxycholate, 1% Triton X-100, 10 mM Tris, pH 7.4, 1 mM Na_3_VO_4_, 10 µg/ml aprotinin, 10 µg/ml leupeptin, 1 mM PMSF) by 100 mg tissue/ml RIPA. Lysates were centrifuged at 23,700 g at 4°C for 15 min, and the supernatants collected. Protein concentrations were determined as above.

### Assay for binding of DIG-labeled Vg to various ligands

LPS, LTA, PGN, β-1,3-glucan and laminarin (all from Sigma) were individually dissolved in re-distilled water, giving concentrations of 40 µg/ml, and a volume of 50 µl (2 µg) of each solution was applied to a 96-well microplate and air-dried overnight at room temperature. The plates were incubated at 60°C for 30 min to fix the ligands, and the wells were each blocked with 200 µl of 1 mg/ml BSA in 10 mM PBS (pH 7.4) at 37°C for 2 h. After washing four times with 200 µl of 10 mM PBS supplemented with 1% Tween-20, 50 µl of DIG-labeled Vg solutions with different concentrations of DIG-labeled Vg (0, 1.25, 2.5, 5, 10 and 15 µg/ml) was added into the wells. After incubation at room temperature for 3 h, the wells were each rinsed four times with 200 µl of 10 mM PBS supplemented with 1% Tween-20, 100 µl of anti-DIG-Fabs (Roche) diluted at 1∶1,000 with 10 mM PBS (pH 7.4) containing 1% dry milk powder was added into the wells. The wells were incubated at 37°C for 2 h, washed as above, and then reacted with 75 µl of 0.4 mg/ml O-phenylenediamine (OPD; Amresco) in 51.4 mM Na_2_HPO_4_, 24.3 mM citric acid and 0.045‰ H_2_O_2_ (pH 5.0) at 37°C for 20 min. Subsequently, 25 µl of 2 mM H_2_SO_4_ was added into each well to terminate the reactions, and absorbance at 492 nm was monitored by a microplate reader (GENios Plus, Tecan). For controls, BSA, serum proteins (SPs) and TMPs of *H. otakii* at the same concentrations were treated similarly.

The ELISA experiments were performed in triplicate, and repeated three times. Statistical calculations were performed using the computer program SPSS 13.0. The statistical significance of difference between mean values was determined by Student's two-tailed *t*-tests, and difference at *p*<0.05 was considered significant. All data were expressed as mean±SEM.

### Isolation of head kidney-derived macrophages and red blood cells

The head kidney of teleostean fish is considered to be the functional and structural homologue of mammalian bone marrow [46], [47]. To procure macrophage population, head kidneys were dissected out from 3 fishes *H. otakii* aseptically, and placed in 10 mM PBS (pH 7.4) containing 300 U/ml penicillin and 300 µg/ml streptomycin. After washing three times with 10 mM PBS with 300 U/ml penicillin and 300 µg/ml streptomycin, the tissues were cut into 1 mm^3^ pieces, gently passed through an 80 µm sterile steel mesh, and rinsed with Leibovitz's L-15 medium (pH 7.2; Gibco) supplemented with 100 U/ml penicillin, 100 µg/ml streptomycin and 2% foetal calf serum (FCS; Gibco). Cell suspensions were again gently passed through a 15 µm sterile steel mesh, and loaded onto a discontinuous gradient consisting of 5 ml of 45% Percoll (Pharmacia) overlaid with 5 ml of 31% Percoll. After centrifugation at 400 g at 4°C for 30 min, the macrophage fraction was collected from 31–45% interface. Macrophage cells were washed three times with Leibovitz's L-15 medium (pH 7.2) supplemented with 100 U/ml penicillin, 100 µg/ml streptomycin and 0.1% FCS, harvested by centrifugation at 200 g at 4°C for 5 min, and suspended in 2 ml Leibovitz's L-15 medium supplemented with 100 U/ml penicillin, 100 µg/ml streptomycin and 5% FCS. The concentration of cells was established by directly counting the cells in the cell suspension in a Burker cell counter, and adjusted to 2×10^7^ cells/ml with Leibovitz's L-15 medium, and the cell viability was assessed by trypan blue exclusion. The resulting cell suspension was stored at 4°C, and used for the following experiments within 2 h.

To obtain red blood cells, a fish *H. otakii* with a weight of about 500 g was anaesthetized, surface-sterilized with alcohol and bled into anti-coagulant solution (10 mM PBS, 600 IU/ml heparin sodium salt, pH 7.4). The plasma was centrifugated at 200 g at 4°C for 5 min. After washing three times with 10 mM PBS, the red blood cells were suspended in Leibovitz's L-15 medium and adjusted to a density of 2×10^7^ cells/ml.

### Labeling microbes with FITC

Labeling microbes with FITC was carried out as described previously [48]. The densities of *E. coli*, *S. aureus* and *P. pastoris* were all adjusted to 5×10^8^ cells/ml, and the microbes were heat-inactivated at 60°C for 15 min. Aliquots of 1 ml of the heat-inactivated samples were mixed with 100 µl of 1 mg/ml FITC in DMSO, and incubated for 30 min in dark with gentle shaking at room temperature. After washing three times with 10 mM PBS (pH 7.4), the FITC-labeled microbes were harvested by centrifugation at 3,000 g at room temperature for 15 min, re-suspended in 1 ml of 10 mM PBS (pH 7.4), and stored at −20°C until used.

### Assay for effects of Vg on phagocytosis

To test the effects of Vg on phagocytosis, aliquots of 300 µl macrophage suspensions were mixed in 1.5 ml eppendorff tubes with 200 µl of 400 µg/ml Vg and 300 µl of FITC-labeled microbe suspensions. The mixtures were incubated at room temperature for 60 min and 105 min, shaking every 5 min to prevent sedimentation of macrophages and microbes, and the phagocytosis process was stopped by addition of 500 µl ice-cold 10 mM PBS (pH 7.4), followed immediately by centrifugation at 200 g at 4°C for 5 min to separate macrophages from non-phagocytosed microbes. The macrophage pellets were re-suspended in 200 µl of 10 mM PBS (pH 7.4), and a volume of 60 µl of the macrophage suspensions was sampled to make smears for microscopic examination under an Olympus BX51 fluorescence microscope. For each example, at least 100 macrophages were observed. The number of macrophages with phagocytosed microbes and the number of microbes in each macrophage were recorded. The phagocytic ability (PA) was defined as the percentage of macrophages with one or more engulfed microbes within the total cell population, and the phagocytic index (PI) as the number of engulfed microbes per cell. For controls, phagocytosis assay was also similarly performed in the presence of BSA instead of Vg and in the absence of Vg.

The phagocytosis experiments were performed in triplicate, and repeated three times. The number of macrophages with phagocytosed microbes and the number of microbes in each macrophage were subjected to statistical evaluation with Student's two-tailed *t*-tests, and difference at *p*<0.05 was considered significant. All data were expressed as mean±SEM.

### Assay for binding of FITC-labeled Vg to macrophages

To test the binding of Vg to macrophages, aliquots of 300 µl macrophage suspensions were mixed in 1.5 ml eppendorff tubes with 200 µl of 400 µg/ml of FITC-labeled Vg. The mixtures were incubated at room temperature for 30 min, shaking every 5 min, and then centrifuged at 200 g at room temperature for 5 min to separate the macrophages from free FITC-labeled Vg. The macrophage pellets were re-suspended in 200 µl of 10 mM PBS (pH 7.4), and a volume of 60 µl of cell suspensions was sampled to make smears for microscopic examination under an Olympus BX51 fluorescence microscope. For control, red blood cells were used, and treated similarly.
